# Significance of a reduction in HCV RNA levels at 4 and 12 weeks in patients infected with HCV genotype 1b for the prediction of the outcome of combination therapy with peginterferon and ribavirin

**DOI:** 10.1186/1471-2334-12-324

**Published:** 2012-11-27

**Authors:** Hidenori Toyoda, Takashi Kumada, Noritomo Shimada, Koichi Takaguchi, Tatsuya Ide, Michio Sata, Hiroyuki Ginba, Kazuhiro Matsuyama, Namiki Izumi

**Affiliations:** 1 Department of Gastroenterology, Ogaki Municipal Hospital, Ogaki, Japan; 2 Division of Gastroenterology and Hepatology, Shinmatsudo Central General Hospital, Matsudo, Japan; 3 Department of Internal Medicine, Kagawa Prefectural Central Hospital, Takamatsu, Japan; 4 Department of Digestive Disease Information and Research, Kurume University School of Medicine, Kurume, Japan; 5 Department of Life Cycle Management, Roche Diagnostics Japan K.K, Tokyo, Japan; 6 Division of Gastroenterology and Hepatology, Musashino Red Cross Hospital, Musashino, Japan

**Keywords:** Chronic hepatitis C, Peginterferon, Ribavirin, Reduction in HCV RNA levels, Four and twelve weeks, Baseline factors, Response-guided therapy, Extended treatment

## Abstract

**Background:**

The importance of the reduction in hepatitis C virus (HCV) RNA levels 4 and 12 weeks after starting peginterferon (PEG-IFN) and ribavirin combination therapy has been reported to predict a sustained virologic response (SVR) in patients infected with HCV genotype 1. We conducted a multicenter study to validate this importance along with baseline predictive factors in this patient subpopulation.

**Methods:**

A total of 516 patients with HCV genotype 1 and pretreatment HCV RNA levels ≥5.0 log_10_ IU/mL who completed response-guided therapy according to the AASLD guidelines were enrolled. The reduction in serum HCV RNA levels 4 and 12 weeks after starting therapy was measured using real-time PCR, and its value in predicting the likelihood of SVR was evaluated.

**Results:**

The area under the receiver operating characteristics (ROC) curve was 0.852 for 4-week reduction and 0.826 for 12-week reduction of HCV RNA levels, respectively. When the cut-off is fixed at a 2.8-log_10_ reduction at 4 weeks and a 4.9-log_10_ reduction at 12 weeks on the basis of ROC analysis, the sensitivity and specificity for SVR were 80.9% and 77.9% at 4 weeks and were 89.0% and 67.2% at 12 weeks, respectively. These variables were independent factors associated with SVR in multivariate analysis. Among 99 patients who showed a delayed virologic response and completed 72-week extended regimen, the area under ROC curve was low: 0.516 for 4-week reduction and 0.482 for 12-week reduction of HCV RNA levels, respectively.

**Conclusions:**

The reduction in HCV RNA levels 4 and 12 weeks after starting combination therapy is a strong independent predictor for SVR overall. These variables were not useful for predicting SVR in patients who showed a slow virologic response and experienced 72-week extended regimen.

## Background

Many investigators have sought to identify factors that can predict the treatment outcome of peginterferon (PEG-IFN) and ribavirin combination therapy in patients infected with HCV genotype 1. Previous studies reported baseline host and viral factors that are associated with the treatment outcomes. The genetic polymorphisms near the *IL28B* gene (rs12979860 or rs8099917) reportedly constitute a host factor that is strongly associated with treatment outcome [1-5], and studies from Japan have reported that amino acid substitutions at residue 70 of the HCV core region and residues 2209–2248 of the NS5A region of HCV (i.e., interferon sensitivity-determining region, ISDR) are viral factors associated with treatment outcome in patients infected with HCV genotype 1 [6-10]. In addition to the baseline predictive factors, the response to HCV during therapy, i.e., the changes in serum HCV RNA levels after initiation of therapy, has also been shown to be an important predictor of treatment outcome [11-14]. Especially, the disappearance or the reduction in serum HCV RNA levels at 4 and 12 weeks after starting therapy have been reported to be important, therefore, rapid virologic response (RVR) or early virologic response (EVR) defined at 4 and 12 weeks after starting therapy, respectively, is a pivotal criteria in predicting treatment response [11-23].

There are adverse effects associated with PEG-IFN and ribavirin antiviral therapy, and the treatment course is costly. For these reasons, it is important to predict the likelihood that a patient will achieve SVR during early stages of therapy with high reliability, in order to prevent unnecessary treatment. This will become increasingly important with the emergence of new antiviral drugs against HCV [24-28]. In the present study, we conducted a multicenter cohort study to examine whether the reduction in HCV RNA levels 4 and 12 weeks after starting PEG-IFN and ribavirin combination therapy, along with baseline predictive factors, has any value in predicting SVR.

## Methods

### Patients, treatments, and evaluation of responses

The inclusion criteria for this multicentre study were (i) infection with HCV genotype 1 without co-infection with hepatitis B virus or human immunodeficiency virus; (ii) pretreatment HCV RNA levels ≥5.0 log_10_ IU/mL, based on a quantitative real-time PCR-based method (COBAS AmpliPrep / COBAS TaqMan HCV Test; Roche Molecular Systems: Pleasanton, CA, US.; lower limit of quantification, 1.6 log_10_ IU/ mL: lower limit of detection, 1.2 log_10_ IU/ mL) [29,30]; (iii) standard PEG-IFN and ribavirin therapy according to the American Association for the Study of the Liver Diseases (AASLD) guidelines [31] started between December 2004 and January 2010; (iv) completed treatment regimen of 48- or 72-week duration with virologic outcomes available for evaluation; and (v) 100% medication adherence for both PEG-IFN and ribavirin during the initial 4 weeks of therapy and 80% or more throughout the treatment period. With regard to inclusion criterion (i), this study did not include any patients infected with HCV genotype 1a because this genotype is usually not found in the Japanese general population. With regard to criterion (ii), we focused on patients with pretreatment HCV RNA level ≥5.0 log_10_ IU/mL because the use of ribavirin along with PEG-IFN is not allowed by Japanese National Medical Insurance System for patients with pretreatment HCV RNA levels <5.0 log_10_ IU/mL. With regard to criterion (iv), the treatment duration was determined based on the response-guided therapy according to AASLD guidelines. Patients in whom serum HCV RNA disappeared until 12 weeks after starting therapy (complete EVR) underwent 48-week treatment regimen. Patients in whom serum HCV RNA disappeared after 12 weeks but until 24 weeks after starting therapy (delayed virologic response) underwent 72-week extended treatment regimen. Patients whose treatment was discontinued due to the presence of serum HCV RNA at 24 weeks of therapy (partial responders or null responders as per the AASLD guidelines), or due to viral breakthrough were also included in the study.

**Figure 1:**
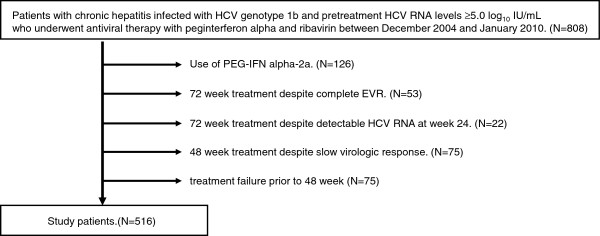
Schematic representation of the study patients.

A total of 808 patients underwent the combination therapy with PEG-IFN and ribavirin between December 2004 and January 2010 in one of the following five Liver Centers: Musashino Red Cross Hospital, Kurume University Hospital, Ogaki Municipal Hospital, Shinmatsudo Central General Hospital, and Kagawa Prefectural Central Hospital. For 126 patients, the treatment regimen consisted of weekly PEG-IFN alpha-2a (Pegasys, Chugai Pharmaceutical, Tokyo, Japan) and daily ribavirin (Copegus, Chugai Pharmaceutical). The other 682 patients were treated with weekly PEG-IFN alpha-2b (Pegintron, MSD Co., Tokyo, Japan) and daily ribavirin (Rebetol, MSD Co.). We excluded patients who had been treated with PEG-IFN alpha-2a and ribavirin in order to avoid the influence of PEG-IFN subtype on the association between viral dynamics and treatment outcome. In 682 patients who received PEG-IFN alpha-2b, 516 patients fulfilled the eligibility criteria and were included for analysis (Figure 1). The doses of PEG-IFN alpfa-2b and ribavirin were adjusted based on the patient’s body weight. Patients ≤ 45 kg were given 60 μg of PEG-IFN alpha-2b weekly, those > 45 kg and ≤ 60 kg were given 80 μg, those > 60 kg and ≤ 75 kg were given 100 μg, those > 75 kg and ≤ 90 kg were given 120 μg, and those > 90 kg were given 150 μg. Patients ≤ 60 kg were given 600 mg of ribavirin daily, those > 60 kg and ≤ 80 kg were given 800 mg, and those > 80 kg were given 1000 mg per day. Dose modifications of PEG-IFN or ribavirin were based on the manufacturer’s recommendations.

SVR was defined as undetectable serum HCV RNA 24 weeks after the end of therapy. A patient was considered to have relapsed when serum HCV RNA levels became detectable between the end of treatment and 24 weeks after completion of therapy, although serum HCV RNA levels were undetectable at the end of therapy. A non-response was defined as detectable serum HCV RNA at 24 weeks after initiation of therapy (i.e., null response or partial non-response according to the AASLD guidelines). RVR was defined as undetectable serum HCV RNA 4 weeks after starting therapy. EVR was defined as the disappearance or a decrease in serum HCV RNA levels by at least 2 log_10_ at 12 weeks after starting therapy. Patients were considered to have a complete EVR if the serum HCV RNA levels were undetectable 12 weeks after starting therapy and a partial EVR if the serum HCV RNA levels were detectable but had decreased by at least 2 log_10_ at 12 weeks of therapy. A non-EVR was defined as a lack of a decrease of HCV RNA by more than 2 log_10_ at 12 weeks when compared to pretreatment levels. Patients were considered to have a delayed virologic response if serum HCV RNA levels became undetectable after 12 weeks but until 24 weeks on treatment.

The study protocol was in compliance with the Helsinki Declaration and was approved by the ethics committee of each participating institution, i.e., the ethics committee of Musashino Red Cross Hospital, the ethics committee of Kurume University Hospital, the ethics committee of Ogaki Municipal Hospital, the ethics committee of Shinmatsudo Central General Hospital, and the ethics committee of Kagawa Prefectural Central Hospital. Prior to initiating the study, written informed consent was obtained from each patient to use their clinical and laboratory data and to analyze stored serum samples.

### Measurements of serum HCV RNA levels, amino acid substitution at residue 70 in the HCV core, amino acid sequence of HCV NS5A-ISDR, and genetic polymorphisms near the IL28B gene

After a patient gave informed consent, serum samples were obtained during the patient’s regular hospital visits, just prior to beginning treatment, and every 4 weeks during the treatment period and the 24-week follow-up period after treatment. Serum samples were stored at −80°C until they were analyzed. HCV RNA levels were measured using a quantitative real-time PCR-based method (COBAS AmpliPrep/ COBAS TaqMan HCV Test) [29,30]. The reduction in HCV RNA 4 and 12 weeks after initiation of therapy was calculated. When calculating the decrease in serum HCV RNA, HCV RNA level was defined as 0 when HCV RNA was undetectable.

Amino acid 70 of the HCV core region and the amino acid sequence of ISDR region (residues 2209–2248 of the NS5A region) were analyzed by direct nucleotide sequencing of each region as previously reported [6,7]. The following PCR primer pairs were used for direct sequencing of the HCV core region:

5′-GCCATAGTGGTCTGCGGAAC-3′ (outer, sense primer),

5′-GGAGCAGTCCTTCGTGACATG-3′ (outer, antisense primer),

5′-GCTAGCCGAGTAGTGTT-3′ (inner, sense primer), and

5′-GGAGCAGTCCTTCGTGACATG-3′ (inner, antisense primer).

The following PCR primers were used for direct sequencing of ISDR:

5′-TTCCACTACGTGACGGGCAT-3′ (outer, sense primer),

5′-CCCGTCCATGTGTAGGACAT-3′ (outer, antisense primer),

5′-GGGTCACAGCTCCCTGTGAGCC-3′ (inner, sense primer), and

5′-GAGGGTTGTAATCCGGGCGTGC-3′ (inner, antisense primer).

When evaluating ISDR, HCV was defined as wild-type when there were 0 or 1 amino acid substitutions in residues 2209–2248 as compared with the HCV-J strain [32], and as non-wild-type when there was more than 1 substitutions.

Genotyping of rs 8099917 polymorphisms near the *IL28B* gene was performed using the TaqMan SNP assay (Applied Biosystems, Carlsbad, CA) according to the manufacturer's guidelines. A pre-designed and functionally tested probe was used for rs8099917 (C__11710096_10, Applied Biosystems). Genetic polymorphism of rs8099917 reportedly corresponds to rs12979860 in more than 99% of individuals of Japanese ethnicity [33]. The TT genotype of rs8099917 corresponds to the CC genotype of rs12979860, the GG genotype of rs8099917 corresponds to the TT genotype of rs12979860, and the TG heterozygous genotype of rs8099917 corresponds to the CT of rs12979860.

### Statistical analyses

Quantitative values are reported as medians and ranges. Differences in percentages between groups were analyzed with the chi-square test. Differences in mean quantitative values were analyzed by the Mann–Whitney U test. The receiver-operating characteristics (ROC) analyses were performed to determine the cut-offs of the reduction in HCV RNA levels at 4 and 12 weeks after starting therapy to evaluate the sensitivity, specificity, positive predictive value (PPV), negative predictive value (NPV), and accuracy for predicting SVR. Univariate and multivariate analyses using a logistic regression model were performed to identify factors that predict SVR. The factors that are potentially associated with SVR were included in the analyses, i.e., age, sex, body mass index (BMI), serum alanine aminotransferase activity, serum gamma-glutamyl transpeptidase level, total-cholesterol levels, neutrophil count, hemoglobin, platelet count, grade of activity and fibrosis of the liver, pretreatment HCV RNA levels, reduction in HCV RNA levels 4 and 12 weeks after starting therapy, amino acid substitution at residue 70 in the HCV core (arginine vs. glutamine or histidine), amino acid mutations in ISDR (non-wild-type vs. wild-type), and genetic polymorphisms near the *IL28B* gene (rs8099917, genotype TT vs. genotype TG or GG). Data analyses were performed using StatFlex statistical software, version 6 (Artech Co., Ltd., Osaka, Japan). All *p* values were two-tailed, and *p* < 0.05 was considered statistically significant.

## Results

### Patient characteristics and treatment outcome

The characteristics of the patients are shown in Table 1. Genotyping of rs8099917 near the *IL28B* gene was performed in 396 patients. Amino acid substitutions at residue 70 in the HCV core region were measured in 361 patients. Amino acid sequences in the ISDR were evaluated in 416 patients. Among 516 patients who were included in the analysis, treatment was completed at 48 weeks in 268 patients who underwent the standard regimen because they showed complete EVR. Treatment was extended from 48 weeks to 72 weeks in 99 patients who yielded delayed virologic response. Treatment was discontinued until 48 weeks in 149 patients because serum HCV RNA remained positive 24 weeks after starting therapy (partial response or null response), or because patients experienced viral breakthrough during therapy.

**Table 1:** Characteristics of study patients

Age (years), median (range)	60.0 (20.0–80.0)
Sex (male/female) (%)	245 (47.5)/ 271 (52.5)
Body weight (kg), median (range)	58.0 (36.35–107.6)
BMI, median (range)	22.7 (15.8–37.0)
Prior treatment for HCV (no/yes) (%)	359 (69.6)/ 157 (30.4)
Initial dose of PEG-IFN (μg), median (range)	80.0 (40.0–150.0)
Initial dose of ribavirin (mg), median (range)	600 (400–1000)
Pretreatment HCV RNA levels (log^10^ IU/mL), median (range)	6.1 (5.0–7.7)
Platelet count (×10^3^/μL)	161 (43–352)
Hemoglobin (g/dL)	13.9 (9.7–17.9)
Neutrophil count (/μL)	2489 (578–7480)
Alanine aminotransferase (IU/L)	47 (10–485)
LDL-cholesterol (mg/dL)	99 (25–226)
Total-cholesterol (mg/dL)	171 (29–325)
γ-glutamyl transpeptidase (IU/L)	34.5 (7.0–579)
Alfa fetoprotein (ng/mL)	5.0 (0.8–584)
Fibrosis score (F1/F2/F3/F4) (%)	208(45.9)/139(30.7)/69(15.2)/37(8.2)
Activity score (A1/A2/A3/A4) (%)	258(56.1)/178(38.7)/24(5.2)/0(0)
Genetic polymorphisms of rs8099917 (TT/GG or TG) (%)	288 (72.7)/ 108(27.3)
Amino acid at residue 70 of HCV core (arginine/glutamine or histidine) (%)	242 (67.0)/ 119 (33.0)
Amino acid sequence of ISDR (non-wild-type/wild-type) (%)	110 (26.4)/ 306 (73.6)

*BMI*, body mass index; *HCV*, hepatitis C virus; *PEG-IFN*, peginterferon; *ISDR*, interferon sensitivity-determining region.

(N = 516).

As a final outcome, 272 patients (52.7%) achieved SVR, 90 patients (17.5%) relapsed, and 128 patients (24.8%) had a non-response (48 patients with partial response and 80 patients with null-response). Viral breakthrough was observed in 26 patients (5.0%). The rate of SVR was 79.9% (214 of 268 patients) among patients with complete EVR in whom treatment was completed at 48 weeks and 58.6% (58 of 99 patients) among patients with delayed virologic response who underwent the extended 72-week regimen.

### Baseline factors affecting SVR in all patients who underwent response-guided therapy according to AASLD guidelines

In all patients who underwent treatment according to the AASLD guidelines, the rate of SVR was significantly higher in patients with the TT genotype of rs8099917 near the *IL28B* gene (179 of 288 patients [62.3%] with TT genotype vs. 15 of 108 patients [13.9%] with TG/GG genotype, *p* < 0.0001). In addition, SVR rate was significantly higher in patients with HCV with arginine at residue 70 in the HCV core region (145 of 242 patients [59.9%] with arginine vs. 34 of 119 patients [28.6%] with glutamine or histidine, *p* < 0.0001). SVR was significantly higher in patients with HCV with non-wild type ISDR (75 of 110 patients [68.2%] with non-wild-type ISDR vs. 139 of 306 patients [45.4%] with wild-type ISDR, *p* < 0.0001). SVR was significantly higher in patients with pretreatment HCV RNA levels <6.0 log_10_ IU/mL (127 of 199 patients [63.8%] with pretreatment HCV levels <6.0 log_10_ IU/mL vs. 145 of 317 patients [45.7%] with pretreatment HCV RNA levels ≥6.0 log_10_ IU/mL, *p* < 0.0001).

### Association between reduction of serum HCV RNA levels 4 and 12 weeks after starting therapy and SVR in all patients who underwent response-guided therapy according to the AASLD guidelines

**Figure 2:**
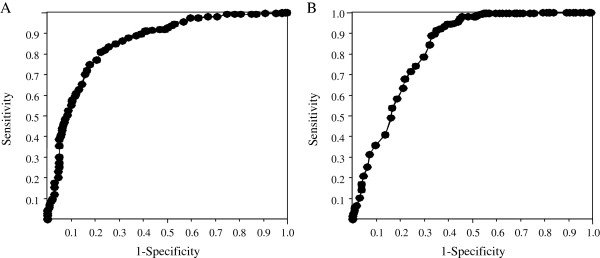
**The receiver operating characteristics (ROC) analysis for the prediction of the sustained virologic response to combination therapy with peginterferon alpha-2b and ribavirin according to the reduction in serum HCV RNA levels in all patients who underwent response-guided therapy based on the AASLD guidelines.A**) According to the reduction in serum HCV RNA levels 4 weeks after starting therapy. The area under the ROC curve was 0.852. **B**) According to the reduction in serum HCV RNA levels 12 weeks after starting therapy. The area under the ROC curve was 0.826.

The ROC analysis was performed in 516 patients who underwent the response-guided therapy according to the AASLD guidelines in order to evaluate the association between the reduction in serum HCV RNA levels 4 and 12 weeks after starting therapy and SVR (Figure 2). The area under the ROC curve was 0.852 and the best cut-off was calculated as 2.8 log_10_ IU/mL, when evaluated with the reduction of serum HCV RNA levels 4 weeks after starting therapy. The rate of SVR was significantly higher in patients with greater than 2.8-log_10_ reduction at 4 weeks (220 of 274 patients [80.3%] with > 2.8-log_10_ reduction vs. 52 of 242 patients [21.5%] with ≤ 2.8-log_10_ reduction, *p* < 0.0001). The sensitivity, specificity, PPV, NPV, and accuracy were 80.9%, 77.9%, 80.3%, 78.5%, and 79.5%, respectively, at this cut-off level. When evaluated with the reduction of serum HCV RNA levels 12 weeks after starting therapy, the area under the ROC curve was 0.826 and the best cut-off was calculated as 4.9 log_10_ IU/mL. The rate of SVR was significantly higher in patients with greater than 4.9-log_10_ reduction at 12 weeks (242 of 321 patients [75.4%] with > 4.9-log_10_ reduction vs. 30 of 194 patients [15.5%] with ≤ 4.9-log_10_ reduction, *p* < 0.0001). The sensitivity, specificity, PPV, NPV, and accuracy were 89.0%, 67.2%, 75.4%, 84.5%, and 78.7%, respectively, at this cut-off level.

A multivariate analysis showed that the reductions in serum HCV RNA levels at 4 and 12 weeks after starting therapy were independent factors associated with SVR, along with pretreatment HCV RNA levels, platelet counts, polymorphisms of rs8099917 near the *IL28B* gene, and amino acid mutations in the HCV NS5A-ISDR (Table 2).

**Table 2:** Univariate and multivariate analyses for sustained virologic response to the combination therapy with peginterferon and ribavirin in patients who underwent response guided therapy according to the AASLD guidelines

	**Univariate analysis**	Multivariate analysis*	**Odds ratio (95% confidence interval)**
Age (years)	< 0.001	N.S.	
Sex (male/female)	0.005	N.S.	
BMI, median (range)	N.S.		
Prior treatment for HCV (no/yes)	N.S.		
Pretreatment HCV RNA levels (log_10_ IU/mL), (≤6.0 vs. 6.0<)	0.015	0.013	2.235 (1.189-4.203)
Platelet count (×10^3^/μL)	< 0.001	0.011	1.007 (1.002-1.013)
Hemoglobin (g/dL)	0.002	N.S.	
Neutrophil count (/μL)	0.003	N.S.	
Alanine aminotransferase (IU/L)	N.S.		
Total-cholesterol (mg/dL)	0.001	N.S.	
γ-glutamyl transpeptidase (IU/L)	0.014	N.S.	
Fibrosis score (F1 or F2/F3 or F4)	< 0.001	N.S.	
Activity score (A1 or A2/A3 or A4)	0.002	N.S.	
Genetic polymorphisms of rs8099917 (TT/GG or TG)	< 0.001	< 0.001	5.782 (2.298-14.552)
Amino acid at residue 70 of HCV core (arginine/glutamine or histidine)	< 0.001	N.S.	
Amino acid sequence of ISDR (non-wild-type/wild-type)	< 0.001	0.038	2.077 (1.041-4.147)
Reduction of HCV RNA [Pre - 4 week] (log_10_ IU/mL), (≤2.8 vs. 2.8<)	< 0.001	< 0.001	3.911 (1.935-7.908)
Reduction of HCV RNA [Pre - 12 week] (log_10_ IU/mL), (≤4.9 vs. 4.9<)	< 0.001	0.013	2.578 (1.220-5.448)

*Multivariate analysis was performed on 314 patients in whom all variables were available.

(N = 516).

### Association between reduction of serum HCV RNA levels 4 and 12 weeks after starting therapy and SVR in patients with delayed virologic response who underwent an extended 72-week regimen according to response-guided therapy

**Figure 3:**
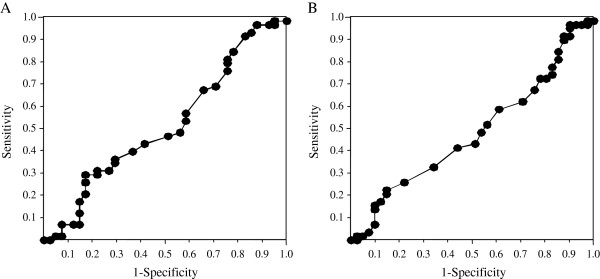
**The receiver operating characteristics (ROC) analysis for the prediction of the sustained virologic response to combination therapy with peginterferon alpha-2b and ribavirin according to the reduction in serum HCV RNA levels in patients with delayed virologic response who underwent an extended 72-week regimen according to response-guided therapy.A**) According to the reduction in serum HCV RNA levels 4 weeks after starting therapy. The area under the ROC curve was 0.516. **B**) According to the reduction in serum HCV RNA levels 12 weeks after starting therapy. The area under the ROC curve was 0.482.

The ROC analysis was performed in 99 patients with delayed virologic response who underwent an extended 72-week treatment regimen according to the response-guided therapy of the AASLD guidelines to evaluate the association between reduction in serum HCV RNA levels 4 and 12 weeks after starting therapy and SVR (Figure 3). The area under the ROC curve was 0.516 and the best cut-off was calculated as 2.3 log_10_ IU/mL, when evaluated with the reduction of serum HCV RNA levels 4 weeks after starting therapy. There was no significant difference in the rate of SVR according to the reduction at 4 weeks (21 of 33 patients [63.6%] with > 2.3-log_10_ reduction vs. 37 of 66 patients [56.1%] with ≤ 2.3-log_10_ reduction, *p* = 0.6120). The area under the ROC curve was 0.482 and the best cut-off was calculated as 5.1 log_10_ IU/mL, when evaluated with the reduction of serum HCV RNA levels 12 weeks after starting therapy. There was no significant difference in the rate of SVR according to the reduction at 12 weeks (24 of 42 patients [57.1%] with > 5.1-log_10_ reduction vs. 34 of 57 patients [59.6%] with ≤ 5.1-log_10_ reduction, *p* = 0.9634).

## Discussion

Several previous studies have reported that patients who achieved RVR, in whom serum HCV RNA levels become undetectable 4 weeks after starting the therapy, had a high likelihood of achieving SVR [15-18]. However, there are relatively few patients infected with treatment-resistant HCV genotype 1 who achieve RVR. A considerable percentage of patients achieve SVR even without RVR. Therefore, RVR has high specificity but low sensitivity for predicting SVR. Previous studies from Asia evaluated the predictive value of the degree of reduction in serum HCV RNA levels 4 weeks after starting therapy, in addition to RVR [19-21]. However, the number of patients in these studies was small and the analyses were not sufficient to form reliable conclusions.

In the present study, we evaluated the ability of a decrease in serum HCV RNA levels 4 weeks after starting therapy to predict the likelihood of SVR as a final outcome in Japanese patients infected with HCV genotype 1b, based on the data from a large, multi-institution study. The ROC analyses showed that a reduction in serum HCV RNA levels 4 week after starting therapy was strongly associated with SVR, and its predictive value was higher than that of a reduction in serum HCV RNA levels 12 weeks after starting therapy, with higher area under the ROC curve and accuracy. Multivariate analyses including baseline factors that were associated with SVR revealed that the reductions of HCV RNA level at both 4 and 12 weeks after starting therapy were independent factors associated with SVR, and the reduction at 4 weeks had a second strongest impact for SVR, following genetic polymorphisms of rs8099917 near *IL28B* gene.

The important novelty from this study is that the reductions of HCV RNA level 4 and 12 weeks after starting therapy had no predictive value for SVR when focusing on patients who showed delayed virologic response and underwent the extended 72-week treatment regimen according to the response-guided therapy. This was in contrast to the prediction for SVR in all patients who underwent response-guided therapy. The impact of the reduction of HCV RNA level on the prediction of SVR would decline by the selection of patients based on the delayed virologic response. There were also no baseline factors that were associated with SVR in patients who underwent the extended 72-week treatment (data not shown). Prolonged treatment duration may relieve delayed virologic responders from unfavorable conditions. Further studies will be, therefore, needed to identify predictive factors for SVR in patients with delayed virologic response who underwent the 72-week treatment regimen.

There are several limitations to this study. The data were based on Japanese patients infected with HCV genotype 1b. Therefore, these results should be confirmed in patients of other ethnicities and patients infected with HCV genotype 1a. In addition, the value of the reduction in HCV RNA levels 4 and 12 weeks after starting therapy as predictors of SVR should be evaluated in patients who underwent therapy with PEG-IFN alpha 2a and ribavirin to determine the best cut-off levels with that regimen. Statistically, there were many missing data. We performed complete case analysis without the imputation of missing data for multivariate analysis. Although comparison between cases with and without missing data did not show statistically significant differences for cases characteristics, we cannot rule out that the condition of data missing completely at random does not hold. Furthermore, this resulted in the decrease in the number of patients analyzed in multivariate analysis and might have substantially caused the reduction of statistical power, altering the value of non-significant results. In addition, the study did not perform internal validation. The use of hold-out method or split-group validation was difficult because of the number of study patients. Therefore, the validation in another larger study patients will be required in the future for confirming the results of this study.

## Conclusions

A reduction in HCV RNA levels 4 and 12 weeks after starting therapy indicated likelihoods that patients will achieve SVR as a final outcome of combination therapy for HCV infection when patients underwent the response-guided therapy according to the AASLD guidelines. These reductions in serum HCV RNA levels were not predictive for SVR when focusing on patients who showed delayed virologic response and underwent the extended 72-week regimen.

## Abbreviations

HCV: Hepatitis C virus; PEG-IFN: Peginterferon; SVR: Sustained virologic response; ROC: Receiver operating characteristics; ISDR: Interferon sensitivity-determining region; RVR: Rapid virologic response; EVR: Early virologic response; AASLD: American Association for the Study of the Liver Diseases; BMI: Body mass index; PPV: Positive predictive value; NPV: Negative predictive value.

## Competing interests

The authors declare the following matters.

The authors have not received reimbursements, fees, funding, or salary from an organization that may in any way gain or lose financially from the publication of this manuscript, neither now nor in the future.

The authors have no stocks or shares in an organization that may in any way gain or lose financially from the publication of this manuscript, neither now nor in the future.

The authors are currently applying no patents relating to the content of the manuscript. We have not received reimbursements, fees, funding, or salary from an organization that holds or has applied for patents relating to the content of the manuscript.

The authors do not have any other financial competing interests.

There are no non-financial competing interests to declare in relation to this manuscript.

This study was supported by Roche Diagnostics Japan, K.K. The employment status of H. Ginba and K. Matsuyama did not influence the data and the interpretation of the study.

## Authors’ contributions

Study design: HT, TK, NS, KT, TI, MS, HG, KM, and NI. Treatment of patients and data acquisition: HT, TK, NS, KT, TI, MS, and NI. Data analyses: HG and KM. Manuscript preparation: HT. Read and approval of the final manuscript: HT, TK, NS, KT, TI, MS, HG, KM, and NI. All authors read and approved the final manuscript.

## Pre-publication history

The pre-publication history for this paper can be accessed here:

http://www.biomedcentral.com/1471-2334/12/324/prepub
